# Prone position in patients with acute respiratory distress syndrome

**DOI:** 10.5935/0103-507X.20160066

**Published:** 2016

**Authors:** Mariano Setten, Gustavo Adrián Plotnikow, Matías Accoce

**Affiliations:** 1 Respiratory Therapists Committee, Sociedad Argentina de Terapia Intensiva - Ciudad Autónoma de Buenos Aires, Argentina.; 2 Centro de Educación Médica e Investigaciones Clínicas - CEMIC - Ciudad Autónoma de Buenos Aires, Argentina.; 3 Sanatorio Anchorena - Ciudad Autónoma de Buenos Aires, Argentina.; 4 Hospital de Quemados - Ciudad Autónoma de Buenos Aires, Argentina.; 5 Sanatorio Mater Dei - Ciudad Autónoma de Buenos Aires, Argentina.

**Keywords:** Prone position, Respiratory distress syndrome, acute/complications, Refractory hypoxemia/etiology, Mechanical ventilation, Posición prona, Síndrome de distrés respiratorio agudo/complicaciones, Hipoxemia refractaria/etiología, Ventilación mecánica

## Abstract

Acute respiratory distress syndrome occupies a great deal of attention in intensive care units. Despite ample knowledge of the physiopathology of this syndrome, the focus in intensive care units consists mostly of life-supporting treatment and avoidance of the side effects of invasive treatments. Although great advances in mechanical ventilation have occurred in the past 20 years, with a significant impact on mortality, the incidence continues to be high. Patients with acute respiratory distress syndrome, especially the most severe cases, often present with refractory hypoxemia due to shunt, which can require additional treatments beyond mechanical ventilation, among which is mechanical ventilation in the prone position. This method, first recommended to improve oxygenation in 1974, can be easily implemented in any intensive care unit with trained personnel.

Prone position has extremely robust bibliographic support. Various randomized clinical studies have demonstrated the effect of prone decubitus on the oxygenation of patients with acute respiratory distress syndrome measured in terms of the PaO_2_/FiO_2_ ratio, including its effects on increasing patient survival.

The members of the Respiratory Therapists Committee of the *Sociedad Argentina de Terapia Intensiva* performed a narrative review with the objective of discovering the available evidence related to the implementation of prone position, changes produced in the respiratory system due to the application of this maneuver, and its impact on mortality. Finally, guidelines are suggested for decision-making.

## Abstract (pt)

El síndrome de distrés respiratorio agudo ocupa gran atención en la unidad de cuidados intensivos. A pesar del amplio conocimiento alcanzado sobre la fisiopatología de éste síndrome, el enfoque en la unidad de cuidados intensivos consiste, en gran parte, en un tratamiento de soporte vital y en evitar los efectos secundarios de las terapéuticas invasivas. Si bien, durante los últimos 20 años, se generaron grandes avances en ventilación mecánica con un impacto importante sobre la mortalidad, ésta continúa siendo elevada. Una característica de los pacientes con síndrome de distrés respiratorio agudo, sobre todo los más severos, es la presencia de hipoxemia refractaria debido a la existencia de *shunt*, pudiendo requerir tratamientos adicionales a la ventilación mecánica, entre ellos la ventilación mecánica en decúbito prono. Este método, recomendado para mejorar la oxigenación por primera vez en 1974, puede ser implementado fácilmente en cualquier unidad de cuidados intensivos con personal entrenado.

El decúbito prono tiene un sustento bibliográfico sumamente robusto. Varios ensayos clínicos randomizados han demostrado el efecto del decúbito prono sobre la oxigenación en pacientes con síndrome de distrés respiratorio agudo medida a través de la relación PaO_2_/FiO_2_ e incluso su impacto en el aumento de la sobrevida de estos pacientes.

Los integrantes del Comité de Kinesiología Intensivista de la Sociedad Argentina de Terapia Intensiva realizaron una revisión narrativa con el objetivo de exponer la evidencia disponible en relación a la implementación del decúbito prono, los cambios producidos en el sistema respiratorio por la aplicación de la maniobra y su impacto sobre la mortalidad. Por último, se sugerirán lineamientos para la toma de decisiones.

## INTRODUCTION

Acute respiratory distress syndrome (ARDS) occupies a great deal of attention in intensive care units (ICU), not only because of its mortality rates but also due to the high resource consumption and the long-term functional and neuro-psychological consequences. In the ICU, the focus largely consists of life-supporting treatments and avoiding the side-effects of invasive treatments such as mechanical ventilation (MV), sedation, neuromuscular blocks, and the administration of high oxygen concentrations.^(1)^ Although great advances in MV with a significant impact on mortality have occurred in the past 20 years,^(2,3)^ the incidence continues to be high.^(3-8)^

Patients with ARDS, especially the most severely affected, often present with refractory hypoxemia due to shunt, which can require additional treatments beyond MV, including MV in the prone position (PP). This method, first recommended to improve oxygenation in 1974,^(9)^ is easily implemented in any ICU^(10)^ and has extremely robust bibliographic support. Various randomized clinical trials (RCTs) have demonstrated the beneficial effect of PP on the oxygenation of patients with ARDS,^(11,12)^ including its effects on increasing patient survival.^(11-14)^

## METHODS

A bibliographic search was performed in the PubMed, SciELO, Cochrane, and Lilacs databases using the following MeSH term and keyword combinations: "randomized controlled trial" OR "controlled clinical trial" OR "random" OR "trial" OR "groups" AND "prone position" (MeSH) OR "supine position" (MeSH) OR "patient positioning" (MeSH) OR "prone" OR "proning" OR "prone position" OR "supine" AND "respiratory distress syndrome, adult" (MeSH) OR "acute lung injury" OR "ARDS" OR "respiratory distress syndrome" OR "respiratory failure". Also included was an unpublished abstract (reference 63) due to its inclusion in one of the meta-analyses.

This narrative review attempts to summarize the physiological modifications associated with PP and to review the clinical trials, meta-analyses, and most relevant systematic reviews from recent years, with special emphasis on the impact on mortality. Finally, this review will establish suggested guidelines and working protocols for decision-making and implementation of MV in PP.

### Physiological modifications associated with prone position

**Figure 1:**
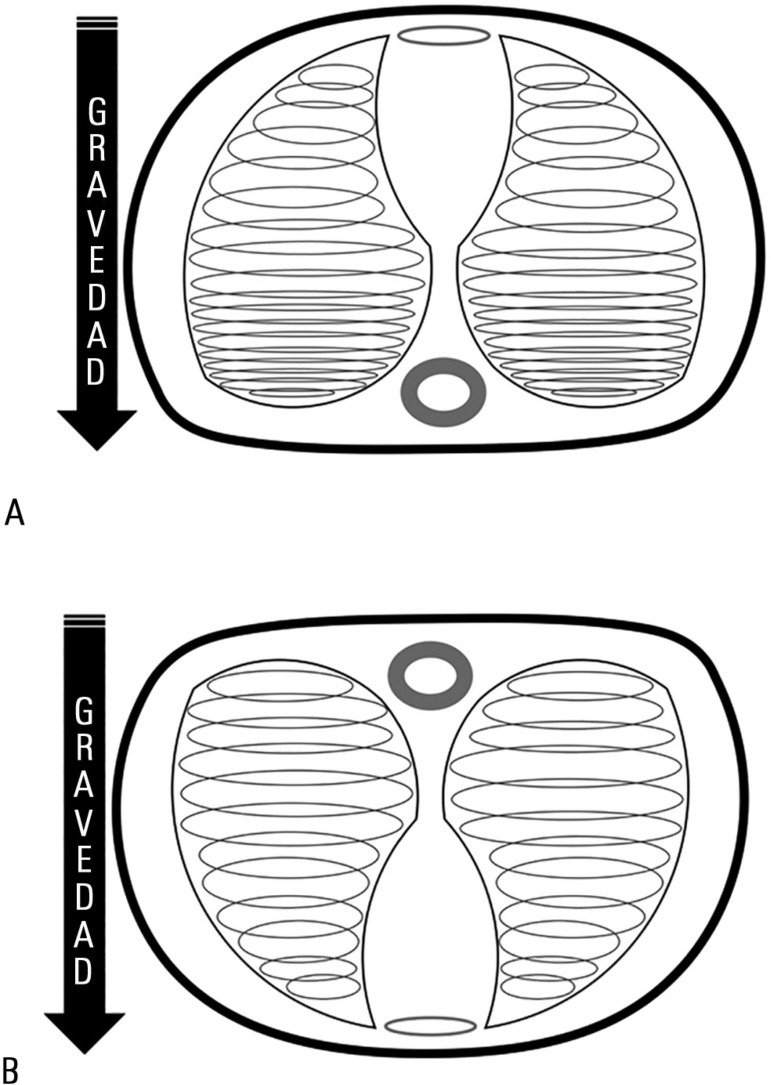
A) Lungs in supine decubitus: Effect of superimposed pressures. Coexistence of relatively normal alveoli with other collapsed but recruitable alveoli, together with other non-recruitable alveolar sectors. B) Lungs in prone decubitus: Effect of prone position on the distribution of pressures in the lung parenchyma and in the homogenization of alveolar ventilation.

In the lungs of patients with ARDS, alveoli in relatively normal condition coexist with others that are collapsed but recruitable, together with other non-recruitable alveolar sectors. This situation produces an increase in lung weight due to edema, generating over-pressure four to five times greater than normal, which precipitates collapse of the most dependent lung regions (compression atelectasis) and increased distension of non-dependent regions due to traction^(8,15,16)^ (Figure 1A).

The displacement of gases into and out of the lungs is determined by a pressure gradient. Elastance of the respiratory system (ERS = ECW + EL) is a function of the elastance values of the chest wall (ECW[ET]) and the lungs (EL[EP]). We can define EL as the difference in transpulmonary pressure over tidal volume (Vt):

- [PAo - esophageal pressure at the end of inspiration] - [PAo - esophageal pressure at the end of expiration]/Vt

(PAo = open airway pressure)

ET can be defined as the difference in esophageal pressure over Vt:

- [Esophageal pressure at the end of inspiration - Esophageal pressure at the end of expiration]/Vt

Changes in patient position are accompanied by changes in elastance, and PP is no exception. Respiratory system elastance can increase, decrease, or remain constant; that is, for any given Vt, the plateau pressure can increase, decrease, or remain constant due to the interaction between the chest wall and the lungs.^(17,18)^

### Lung elastance behavior

In a patient on MV and without diaphragmatic activity, during inspiration, air is directed to non-dependent regions due to collapse of the dependent regions. In the prone position, the availability of the pulmonary parenchyma increases. Collapsed alveoli, potentially recruitable, are reopened, and the inferior lobes (which have a higher quantity of alveoli than the superior lobes) offer higher surface area for diffusion, at once improving ventilatory pressures and decreasing the deformation of fibers (strain) and tension (stress) (Figure 1A and 1B). Prone position varies the pressure gradient distribution in relation to the redistribution of the infiltrated areas, the weight of the cardiac mass (the supine position compresses the left lower lung lobe), variations in EL, and cephalic displacement of the abdomen, which results in more homogenous alveolar ventilation.^(8,12,16,19-26)^

When there is a net provoked alveolar recruitment, EL decreases proportional to the degree of recruitment. If the decrease in EL is similar to the increase in ECW, respiratory system elastance is maintained with no changes. In contrast, if the decrease in EL associated with recruitment is greater than the increase in ECW, the final result will be a decrease in respiratory system elastance.

Increases in stress and strain produce structural changes in the alveoli, including cellular damage, surfactant dysfunction, edema and increased capillary permeability, and biological alterations, such as increased proinflammatory mediators.^(22)^ Decreases in *stress* and *strain* produced by PP can have some influence on these mechanisms and decrease the risk of ventilator-induced damage.^(27)^

Mentzelopoulos et al. have demonstrated that, in patients with severe ARDS, implementation of PP combined with optimization of the positive-end expiratory pressure (PEEP) level post-procedure improves lung volume at the end of expiration, increasing it by approximately 30%, with reductions in elastance and pulmonary resistance. At the same time, PP reduces pulmonary stress (reflected by reduced transpulmonary pressure) and strain (reflected by the relationship between Vt/lung volume at the end of expiration, decreasing from 27% to 33%) compared to Fowler's position.^(28)^

Cornejo et al. evaluated the responses to PP combined with high levels of PEEP (15cmH_2_O) in 24 patients with ARDS. They found that using this strategy improved pulmonary recruitment, as evidenced by decreases in unventilated lung tissue from 501 to 322 grams (p < 0.001) when using 15cmH_2_O for PEEP and from 322 to 290 grams (p = 0.028) when combined with PP. Likewise, this strategy (PP + PEEP 15cmH_2_O), in patients with high recruitment potential, decreased alveolar instability from 4.1 ± 1.9% to 2.9 ± 0.9% (p = 0.003).^(25)^

### Chest wall elastance behavior

The dorsal region of the chest wall is more rigid than the ventral region due to the presence of the spinal column and para-vertebral muscle masses. When a patient is placed in the PP, thoracic expansion is produced mainly in the direction of the abdominal and dorsal regions. In addition to these changes, we may assume that the ventral wall becomes more rigid as a result of the position *per se*, with a resulting increase in ECW. Recalling the foregoing explanation, if EL does not change, the result is an increase in respiratory system elastance secondary to increased ECW.^(29)^

### Prone position and intra-abdominal pressure

Although their behaviors may be unique, we can describe the thoracic and abdominal cavities as two compartments of different volume.^(29)^ The two compartments are occupied by organs of different densities and are separated by the diaphragm. With respect to the difference in chest wall rigidity (the dorsal wall is more rigid than the ventral), both pleural and intra-abdominal pressures would be modified by a change in body position, influenced by the increase in abdominal wall rigidity. Increases in intra-abdominal pressure influence the curvature and position of the diaphragm.^(30)^

In the supine position, the abdominal cavity hydrostatic pressure can be as much as five times higher than that in the thoracic cavity,^(31)^ a difference that increases significantly in obese patients.^(32)^ The causes of ARDS are also associated with syndromes that considerably increase the intra-abdominal pressure, such as abdominal compartment syndrome, which can cause pressures up to 34cmH_2_O.^(33)^ In these conditions, the highest intra-abdominal pressures in supine decubitus correspond to the dorsal regions, where pressure is inexorably transmitted to the pleural space, generating extrinsic compression to the postero-basal pulmonary region. Prone position modifies this situation, and some authors have reported decreased intra-abdominal pressure;^(34)^ in the end, the abdominal wall becomes more rigid, with a resulting increase in intra-abdominal pressure.^(35-37)^

### Changes in the ventilation/perfusion ratio

Describing a lung model in vertical position suggests a ventilation/perfusion ratio (V/Q) based on a "gravitational" hypothesis, which may explain why perfusion is greater in the more dependent regions of the lungs. Studies of PP, both human and experimental, confirm the hypothesis in which the distribution of perfusion shows a non-gravitational gradient. Upon making the non-dependent zones the more perfused and increasing the ventilated lung volume in PP, a notable improvement in the V/Q ratio is produced.^(38-40)^ Other factors influencing this type of perfusion distribution are the fractal architecture of the vessels, greater production of nitric oxide in dorsal zones compared to the ventral, and lower vascular resistance in dorsal zones.^(41-45)^

### Effects of the prone position on hemodynamics

We could suppose that the mere fact of changing the mediastinum's position in the thoracic cavity by placing patients in PP has some hemodynamic effect. In a study of patients without ARDS, the elimination of the weight of the heart from ventral lung zones showed a freeing of a small portion of the lung parenchyma.^(46)^ However, this effect is different in patients with cardiomegaly and congestive heart failure, situations often associated with ARDS, and the improvement in oxygenation upon adopting the PP position is immediate,^(47)^ possibly explained by a greater portion of lung parenchyma freed by this maneuver.^(48)^

However, the specific effects on hemodynamic changes have also been studied via impacts on the right-ventricular ejection fraction,^(49)^ favored due to a decrease in the load and explained by PP. Another study^(50)^ demonstrated an increase in preload and a decrease in postload of the right ventricle and an increase in preload of the left ventricle.

During PP, the pulmonary artery occlusion pressure was also increased, with a decrease in the transpulmonary pressure gradient (difference between the average pulmonary artery pressure and its occlusion pressure), which was associated with "pulmonary vascular dysfunction" and may be associated with an increase in mortality in patients with ARDS.^(51,52)^

Prone position also has an impact on the extravascular lung water index, although its clinical relevance has not been observed.^(53,54)^ While large studies on PP in patients with ARDS have excluded those with hemodynamic instability, patients with myocardial ischemia can be more susceptible to cardiac dysfunction during PP.^(55,56)^

### Studies included for analysis

For the review, the five RCTs considered most relevant were selected (Table 1), in which the attempt was made to show that ventilation in PP in patients with hypoxemia decreases mortality, in addition to six reviews and meta-analyses. Below, we will analyze the results.

**Table 1:** Comparative description of the five most relevant randomized clinical studies selected for review

	Gatinnoni et al., 2001^(11)^	Guerin et al., 2004^(12)^	Mancebo et al., 2006^(13)^	Taccone et al., 2009^(57)^	Guerin et al., 2013^(14)^
Number of patients	304	791	136	342	466
Prone/Supine	152/152	413/378	76/60	168/174	237/229
ALI/ARDS**	6/94	21/31/others	ARDS	ARDS	ARDS
PaO_2_/FiO_2_	127	153	145	113	> 150
Prone duration (hours/day)	7 ± 1.8*	8# (IR 7.7; 9.8)	17#	18 ± 4*	17 ± 3*
Days of pronation	4.7*	4# (IR 2 - 6)	10.1# (IR 0 - 54)	8.4 ± 6*	4 ± 4*
Protective ventilation	No	No	Yes	Yes	Yes
Weaning protocol	No	Yes	Yes	---	Yes
Primary result**	Mortality 10 days	Mortality 28 days	Mortality ICU	Mortality 28 days	Mortality 28 days
	21.1/25	32.4/31.5	43/58	31/32.8	16/32.8
Mortality ICU**	50.7/48	---	---	38.1/42	---
Mortality day 90**	---	43.3/42.2	---	---	23.6/41
Mortality in hospital**	---	---	50/62	---	---
Mortality at six months**	62.5/58.6	---	---	47/52.3	---

ALI - acute lung injury; ARDS - acute respiratory distress syndrome; IR - interquartile range; ICU - intensive care unit.

* Average ± standard deviation;

# Median and interquartile ranges (IR);

** %.

### Results of clinical trials

The first RCT, published in 2001 by the Prone-Supine Study Group,^(11)^ randomized 304 patients with a wide range of severity of acute lung injury. The patients were kept prone for an average of seven hours/day for a maximum of 10 days, but there was no effect on survival. Three years later, Guerin et al.^(12)^ executed a similar multicentric study: patients were kept in PP for approximately eight hours/day until compliance with clinical improvement criteria was met. This study also showed no reduction in mortality.

**Figure 2:**
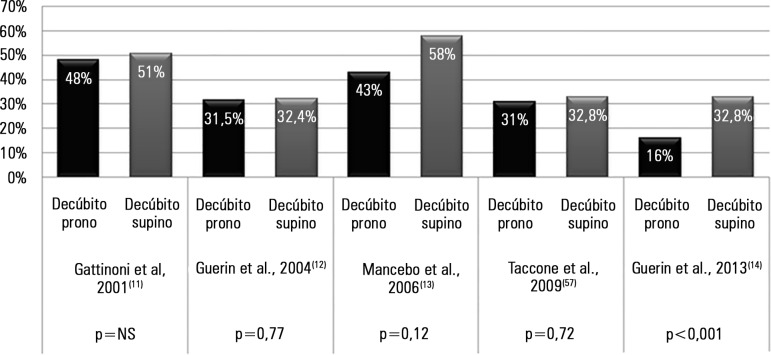
Comparison of the results from various randomized clinical studies related to mortality at day 28 with respect to the use of prone position. NS - not significant.

Two subsequent multicentric RCT attempted to correct some of the deficiencies of the prior studies: they only included patients with ARDS and kept them prone for approximately 20 hours/day. The study performed by Mancebo et al.^(13)^ was ended prematurely, after including only 142 patients, due to recruitment difficulties. The most recent RCT by Taccone et al.^(57)^ (Prone-Supine II Study) included 342 patients and showed a significantly higher frequency of adverse events in patients receiving PP. Neither of the two studies mentioned showed improvements in survival, not even in patients with severe ARDS (Figure 2).

In 2013, the French multicentric RCT PROSEVA Study Group^(14)^ showed a marked improvement in mortality at day 28 (Figure 2): 16% in the prone group (38/237 patients) *versus* 32.8% (75/229 patients) in the supine group (p < 0.001). The study's design had these novel characteristics:

- Use of protective MV (6mL/kg ideal body weight as a starting point, together with plateau pressure < 30cmH_2_O).
- Inclusion with patients with severe ARDS, defined as PaO_2_/FiO_2_ < 150mmHg with PEEP ≥ 5cmH_2_O and FiO_2_ ≥ 60%.
- Stabilization period of 12 - 24 hours prior to randomization, which allowed the selection of patients with ARDS who did not improve solely with recruitment, discarding those with atelectasis or hydrostatic pulmonary edema as significant contributors to the acute hypoxemia.
- Use of neuromuscular blocks on continuous infusion during the initial 48 hours.
- Decoupling of the MV that included standardized interruption of sedation.

### Results of reviews and meta-analyses

As previously mentioned, some reviews and meta-analyses have been published with the objective of analyzing the data from RCTs published on the topic with more representative samples, effecting stratifications according to PaO_2_/FiO_2_ and number of hours/day in the prone position, to elucidate whether subgroups with differences in mortality exist.

Abroug et al.^(58)^ published a meta-analysis that included six RCTs with data from 1372 patients to analyze mortality in the ICU at 28 days as the primary variable. A total of 713 patients were ventilated in PP, with 659 ventilated in the supine position. The secondary variables included changes in PaO_2_/FiO_2_ and the incidence rates of ventilator-associated pneumonia (VAP) and adverse effects of PP. Also analyzed was the duration of stay in the ICU. Ventilation in PP was not associated with an improvement in survival, with a change in mortality of 3% (odds ratio [OR] 0.97, 95% confidence interval [CI] 0.77 - 1.22). Regarding oxygenation, ventilation in PP in this meta-analysis showed a significant improvement in PaO_2_/FiO_2_ (95%CI: 15 - 35, p < 0.00001, I^2^ = 56%). The results of this meta-analysis do not justify the routine use of PP during MV in patients with acute hypoxemic respiratory insufficiency, including acute lung injury and ARDS.

In the same year, another meta-analysis was published by Sud et al.^(10)^ that included more studies with a small number of patients. Their objectives were to evaluate mortality, oxygenation, VAP, duration of MV, and adverse effects. In the primary analysis (10 clinical studies,^(11-13,59-65)^ n = 1486), ventilation in PP did not decrease mortality (relative risk [RR] 0.96, 95%CI: 0.84 - 1.09; p = 0.52). The duration of the prone position was up to 24 hours over one to two days in the short-term studies^(63-65)^ and up to 24 hours a day over more than two days in longer-term studies.^(11-13,59-62)^ In subgroup analysis (short- and long-duration PP), significant mortality differences were likewise not found (RR 0.77, 95%CI: 0.46 - 1.28 and OR 0.97, 95%CI: 0.85 - 1.11, respectively; p = 0.39 for comparison of the two ORs). Ventilation in PP increased the PaO_2_/FiO_2_ by 23 - 34% in the first three days after randomization, measured at the end of the prone period. Post-hoc analysis revealed that the major part of this improvement was produced during the first hour of the prone position. In six studies^(12,13,59-61,66)^ (n = 1026), ventilation in PP reduced the risk of VAP (RR 0.81, 95%CI: 0.66 - 0.99, p = 0.04), without heterogeneity (I^2^ = 0%). In six studies^(11,59-63)^ (n = 504), ventilation in the prone position increased the risk of pressure ulcers (RR 1.36, 95%CI: 1.07 - 1.71; p = 0.01; I^2^ = 0%).

In 2010, Sud et al.^(67)^ published a systematic review and meta-analysis focused on the impact on mortality, hypothesizing that ventilation in PP might reduce mortality in severely hypoxemic patients (PaO_2_/FiO_2_ < 100mmHg), but not in patients with moderate hypoxemia (100mmHg ≤ PaO_2_/FiO_2_ ≤ 300mmHg). The primary variable was mortality in the patient subgroup with PaO_2_/FiO_2_ < 100 mmHg *versus* patients with PaO_2_/FiO_2_ ≥ 100 and ≤ 300mmHg. For each study, mortality was determined on discharge from the hospital or on later follow-up. Secondary results included mortality stratified according to PaO_2_/FiO_2_ but were limited to patients with acute lung injury/ARDS; in all patients, the results also included duration of MV, days off of MV up to day 28, and adverse events. The review included 10 studies^(1,11-13,57,59-62,66)^ (n = 1867; one study^(62)^ included 102 children). Seven^(1,11-13,57,61,62)^ of the ten studies reported mortality stratified by PaO_2_/FiO_2_ and were included for the primary variable. Ventilation in PP significantly reduced mortality in patients with PaO_2_/FiO_2_ < 100mmHg (RR 0.84, 95%CI: 0.74 - 0.96, p = 0.01, n = 555), but not in patients with PaO_2_/FiO_2_ ≥ 100mmHg (RR 1.07, 95%CI: 0.93 - 1.22, p = 0.36, n = 1169). In the severely hypoxemic subgroup, the number of patients necessary to pronate to avoid one death was 11 (95%CI 6 - 50). Post-hoc analyses with variation of cut-offs for PaO_2_/FiO_2_ suggested a decrease in mortality in the most severe subgroup, using a PaO_2_/FiO_2_ cutoff limit of up to approximately 140mmHg. In the first three days after randomization, prone ventilation improved PaO_2_/FiO_2_^(1,11-13,57,61,62,66)^ between 27 and 39% in seven studies. In spite of these improvements, there were no effects on the duration of MV (average difference -0.70 days, 95%CI -2.01 to 0.62 days, p = 0.3; eight studies,^(1,11,12,57,59,60,62,66)^ n = 1588) or in days off of MV up to day 28 (average difference -0.88 days, 95%CI: -2.14 to 0.37 days, p = 0.17; 5 studies^(1,11,57,60,62)^ n = 771). According to this meta-analysis, PP increases the risk of pressure ulcers (RR 1.29, 95%CI: 1.16 - 1.44, p < 0.00001; seven studies,^(11,13,59-62)^ n = 1279), endotracheal tube obstruction (RR 1.58, 95%CI 1.24 - 2.1, p = 0.0002; seven studies^(1,12,57,59,60,62,64)^ n = 1351), and accidental chest tube removal (RR 3.14, 95%CI 1.02 - 9.69, p = 0.05; eight studies,^(1,11,57,59-62,64)^ n = 886, of which only two studies^(11,57)^ reported events).

In the same year, a meta-analysis by Gattinoni et al.^(68)^ included four works^(11-13,57)^ for analysis of the mortality variable and found, as in the meta-analysis by Sud et al.,^(67)^ differences in favor of the prone patient group with severe hypoxemia (PaO_2_/FiO_2_ < 100mmHg).

In 2011, Abroug et al.^(69)^ published a new meta-analysis that focused on a subanalysis of studies prior to 2005 and included seven works^(1,11-13,57,59,61)^ (n = 1675; 862 ventilated in prone from seven to 24 hours/day). Studies published prior to 2006^(11,12,59)^ included 1135 patients with acute lung injury/ARDS, with a short prone duration (less than 17 hours/day), and without protective ventilation. The four most recent studies^(1,13,57,61)^ included only patients with ARDS (n = 540) and applied the prone position for longer times (17 - 24 hours/day), using protective ventilation. In only the most recent four studies^(1,13,57,61)^ (n = 540) that included only patients with ARDS, PP significantly reduced mortality in the ICU (OR 0.71, 95%CI 0.5 - 0.99, p = 0.048; number required to treat = 11; I^2^ = 0%).

In 2014, Beitler et al.^(70)^ published a meta-analysis whose primary variable was mortality at 60 days. This study included seven RCTs^(1,11-13,57,59)^ (n = 2119); 1088 patients were ventilated in the prone position and 1031 in the supine position. To test the *a priori* hypothesis that PP reduces mortality only when high and harmful tidal volumes are avoided, the analysis was stratified according to high Vt (more than 8mL/kg predicted body weight) *versus* low Vt (less than or equal to 8mL/kg). After the stratification, PP was associated with a significant decrease in mortality in the studies that used low tidal volumes (RR = 0.66, 95%CI 0.50 - 0.86, p = 0.002), but not for those using high tidal volumes (RR = 1.00; 95%CI 0.88 - 1.13, p = 0.949). Stratification by Vt substantially reduced heterogeneity (I^2^: from 64% to 11% and 25% in high- and low-Vt models, respectively). The meta-regression demonstrated a dose-response relationship between average basal Vt (mL/kg predicted body weight) and the relation of mortality risk at 60 days in PP. A decrease in the average basal Vt of 1mL/kg was associated with a mortality risk decrease of 16.7% (95%CI 6.1 - 28.3, p = 0.001). Analysis stratified by long- or short-duration prone positioning showed a significant reduction in mortality with long duration (RR = 0.71, IC95% 0.56 - 0.90, p = 0.004). This meta-analysis demonstrates that PP significantly reduces mortality in patients with ARDS when it is used with low Vt.

### Recommendations (Figure 3)

**Figure 3:**
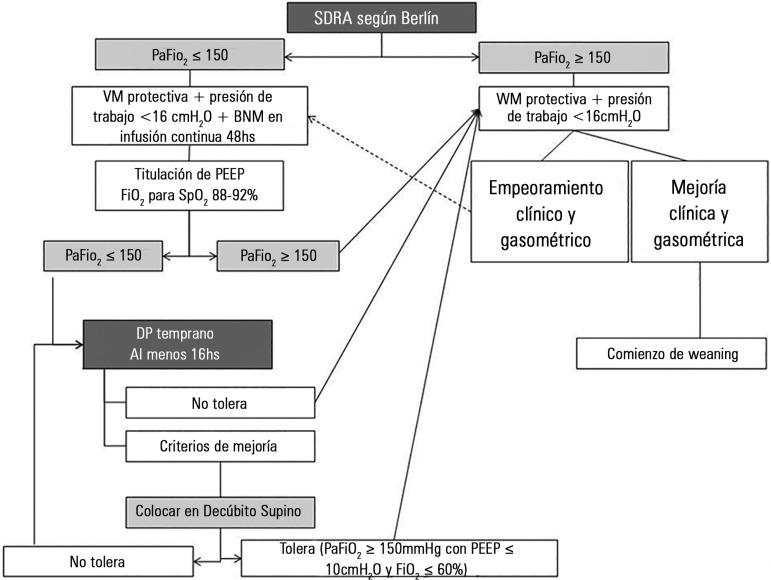
Decision-making algorithm for implementing prone position in patients with acute respiratory distress syndrome. ARDS - acute respiratory distress syndrome; MV - mechanical ventilation; NMB - neuromuscular blockade; PEEP - positive-end expiratory pressure; PP - prone position.

- Define ARDS according to Berlin's definition.
- In ARDS, early intervention with PP is effective (first 24/36 hours from initiation of MV).
- Prior to PP, define the severity of ARDS with a sedated patient, adapted to the MV (RASS - 4/-5), with muscle relaxers (if necessary) on continuous infusion, ventilated using a protective strategy of 6 - 8mL/kg predicted weight of Vt, PEEP ≥ 5cmH_2_O, plateau pressure < 30cmH_2_O, working pressure < 16cmH_2_O and FiO_2_ with a saturation target of 88 - 92%.
- PP offers advantages in terms of survival of patients with relatively severe ARDS (PaO_2_/FiO_2_ ≤ 150mmHg).
- In the majority of cases, a minimum of four people is required to implement PP.
- Protect the areas most subject to decubitus lesions: hips, knees, shoulders, and face.
- Once the maneuver is performed, re-evaluate the PEEP level required.
- PP sessions should ideally be maintained from 16 to 20 hours. During this time, the patient should alternate positions (swimmer's pose).
- PP may be suspended due to positive or negative effects. Positive: PaO_2_/FiO_2_ > 150mmHg for at least four hours in the supine position following the last PP period (with PEEP ≤ 10 cmH_2_O and FiO_2_ ≤ 60%). Negative: deterioration of oxygenation (decrease in PaO_2_/FiO_2_ > 20%) in PP *versus* supine decubitus.
- Consider some of the unanticipated events that may occur during the maneuver and require it to be halted:
  - Accidental extubation.
  - Sustained desaturation (< 85%) or PaO_2_ < 55mmHg with FiO_2_ 100% sustained over five minutes.
  - Cardiac arrest or sustained bradycardia (≤ 30 beats per minute for one minute).
  - Hypotension (< 60mmHg) sustained for five minutes.
  - Any other situation that, according to the treatment team's criteria, is considered a health risk for the patient.

### Maneuver for placing the patient in prone position

At least four operators will be required. One is in charge of the airway, two are in charge of rotating the patient, and one more gives direction and checks catheters, tubes, lines, and probes. The maneuver begins by placing the patient in the lateral decubitus position. Once it is decided which position will be used, the length of any guides, probes, catheters, and tubes the patient has in place should be checked. Shut off nutrition and re-evaluate the hemodynamic situation. The ability to apply protective patches to areas prone to decubitus lesions is also necessary (knees, shoulders, face).

First, move the patient toward the edge of the bed opposite the side onto which they will be turned. The hand on the rotational side should be placed in contact with the ipsilateral buttock (palm-buttock).

For the second step, place the patient in the lateral decubitus position. Check catheters, probes, and tubes, and control hemodynamics.

In the third step, place the patient in the prone position and recheck everything mentioned in the previous step. It is recommended that leg and arm positions be alternated (swimmer's position) to avoid decubitus lesions. This precaution also applies to the face.

## CONCLUSIONS

The prone position is a maneuver that can have a significant impact on respiratory physiology and is useful and attainable for the majority of intensive care units. Supported by robust scientific evidence, its implementation should be considered in a select group of patients who would benefit in terms of mortality. The application of this maneuver should be made a part of protocol and should be performed by trained personnel, adapted to the particulars of each institution.
